# Reusable Medical Isolation Gowns with a Liquid Barrier: Washing Gowns in the Coronavirus Disease 2019 Pandemic Era?

**DOI:** 10.31662/jmaj.2021-0075

**Published:** 2021-12-15

**Authors:** Hiromichi Naito, Kohei Tsukahara, Soshi Takao, Takashi Yorifuji, Atsunori Nakao

**Affiliations:** 1 Department of Emergency, Critical Care, and Disaster Medicine, Okayama University Graduate School of Medicine, Dentistry and Pharmaceutical Sciences, Okayama, Japan; 2 Department of Epidemiology, Okayama University Graduate School of Medicine, Dentistry and Pharmaceutical Sciences, Okayama, Japan

**Keywords:** Coronavirus disease 2019, Personal protective equipment, Waterproof, Reuse

## Abstract

Healthcare providers are at risk of exposure to SARS-CoV-2 via droplets, respiratory secretions, and contact with contaminated surfaces. Personal protective equipment (PPE) is necessary for primary reliable prevention to treat patients with coronavirus disease 2019 (COVID-19). However, PPE shortages have had a significant impact on every medical facility, and outpatient clinics are especially vulnerable to shortages of medical supplies. During the first stage of the pandemic, efforts were made to reduce the use of medical supplies. Guidance and strategies were proposed to ration the use of PPE, including reusing it. However, reuse (wash) of isolation gowns has not been practically promoted despite these suggestions. Further, reusable products may have advantages for economic and ecologic reasons. We developed an adult universally sized, long-sleeved, 100% polyester, reusable/washable gown with liquid barrier protection. The isolation gown can be worn repeatedly through washing and subsequent disinfection, and it can withstand washing in 80°C hot water for 10 min and/or immersion in 0.05%-0.1% sodium hypochlorite for 30 min and then dried. This new gown’s liquid barrier performance is at Association for the Advancement of Medical Instrumentation level 1, even after 20 repeated uses with low cost. The choice of barrier level for gowns should be made based on the risk of contamination. However, the healthcare setting for COVID-19 patients varies greatly with not fully elucidated transmissibility. The newly made reusable isolation gown can be one option for treating COVID-19 patients especially in low-risk settings with economical advantage. Further, preparedness for reuse may have critical implications in extreme shortage. Reconsideration should be focused on reusable gowns with liquid barrier performance and their appropriate use.

Coronavirus disease 2019 (COVID-19), resulting from severe acute respiratory syndrome coronavirus 2 (SARS-CoV-2) infection, has presented as a national emergency in many countries and remains a significant issue. Healthcare providers are at risk of exposure to SARS-CoV-2 via droplets, respiratory secretions, and contact with contaminated surfaces ^(1)^. The COVID-19 pandemic has resulted in escalating cases. Since the beginning of 2020, this has caused unprecedented shortages of medical supplies, including personal protective equipment (PPE), which is necessary for primary reliable prevention. PPE shortages have had a significant impact on every medical facility, and outpatient clinics are especially vulnerable to shortages of medical supplies. In fact, more than half of small outpatient clinics had severe shortages of provisions needed for medical care in Tokyo ^(2)^. During the first stage of the pandemic, efforts were made to reduce the use of medical supplies. Guidance and strategies were proposed to ration the use of PPE, including reusing it ^(3)^. Indeed, practical reuse of “medical masks” with appropriate decontamination/sterilization procedures was reported ^(4)^. In severe shortage at 2020, even quickly manufactured alternative use was reported for “isolation gowns,” which were made from plastic bags. However, reuse (wash) of isolation gowns has not been practically promoted despite these suggestions. The risk of severe shortage seems decreased. Nevertheless, preparations for medical supply shortage are a critical concern. Further, reusable products may have advantages for economic and ecologic reasons.

The choice of barrier level for gowns should be made based on the risk of contamination. The standards for liquid barrier performance and classification of protective apparel for healthcare providers are set by the Association for the Advancement of Medical Instrumentation (AAMI), with four levels of protection ^(5)^. Generally, healthcare providers who are at risk for direct contact with blood, body fluids, and/or other potentially infectious materials such as those encountered during surgeries need a higher level of barrier protection, such as AAMI levels 3-4, whereas for healthcare providers with a low or minimal risk of bodily fluid exposure, AAMI level 1-2 gowns may be used. The healthcare setting for COVID-19 patients varies greatly with not fully elucidated transmissibility. This seems to make healthcare providers who are at risk willing to use products providing a stronger liquid barrier (disposable) instead of reusable apparel. Because classical cotton-made apparel has water absorbency, the Centers for Disease Control and Prevention points out the potential capability of reusing polyester or polyester-cotton fabrics, which can be manufactured into waterproof products. Gowns with liquid barrier performance with an assured AAMI level can be one clue for the practical reuse of medical isolation gowns.

At the time of extreme shortage of PPE, an adult universally sized, long-sleeved, 100% polyester, reusable/washable medical isolation gown with liquid barrier protection has been fabricated (KANKO Co., Okayama, Japan) by a company that originally makes school uniforms and wears. The isolation gown can be worn repeatedly through washing and subsequent disinfection, and it can withstand washing in 80°C hot water for 10 min and/or immersion in 0.05%-0.1% sodium hypochlorite for 30 min and then dried ^(6)^. This new gown’s liquid barrier performance was tested (Nissenken Quality Evaluation Center Co., Tokyo, Japan) using impact penetration test/hydrostatic pressure test and confirmed at AAMI level 1, even after 20 repeated uses, washes, and disinfections. This reusable gown costs approximately $3/use ($50 for one reusable gown with 20 times use; $0.5 for washing with sodium hypochlorite). The cost of disposable gowns varies greatly. However, a long-sleeved assured AAMI level disposable gown costs $9/use ($5 for one disposable gown; $4 for disposal as medical infectious waste) in our medical settings. Reusable gowns seem to have disadvantages in the washing/disinfecting process, which may endanger healthcare workers, and extra laundry space may be needed. Although we confirmed that these new reusable gowns are very tough and confer a considerable waterproof level of protection, fractures must be checked during the washing process. These reusable gowns are being used in outpatient clinics with suspected COVID-19 patients or low risk for fluid exposure during SARS-CoV-2-positive patient care.

Risks and barrier levels of isolation gowns needed to treat COVID-19 patients should be further evaluated. Appropriate reuse of isolation gowns may provide economic and ecological benefits. Moreover, preparedness for reuse may have critical implications in extreme shortage. Reconsideration should be focused on reusable gowns with liquid barrier performance and their appropriate use.

## Article Information

### Conflicts of Interest

The reusable gowns for the study were kindly provided by KANKO Co. (Okayama, Japan), without any financial support. They have no input to the study.

### Acknowledgement

We would like to thank Christine Burr for editing the manuscript.

### Author Contributions

HN, KT, ST, TY, and AN contributed to the conception, data acquisition, analysis, and writing of the manuscript. All authors read and approved the final manuscript.

### Approval by Institutional Review Board (IRB)

IRB Approval was waived.
